# Haplotype-based nested association mapping for net form net blotch response in Australian barley

**DOI:** 10.1007/s00122-026-05328-0

**Published:** 2026-08-18

**Authors:** Muhammad Nadeem, Dan Liu, Lisle Snyman, Lee Hickey, Eric Dinglasan

**Affiliations:** 1 Queensland Alliance for Agriculture and Food Innovation, The University of Queensland, St Lucia, QLD Australia; 2 Department of Primary Industries, Hermitage Research Facility, Warwick, QLD Australia

## Abstract

**Key message:**

**Haplotype-based nested association mapping conducted in Australian barley identified 30 haploblocks for resistance/susceptibility to net form of net blotch. Stacking multiple resistance haplotypes significantly reduced disease severity.**

**Abstract:**

Net form net blotch (NFNB), caused by *Pyrenophora teres f. teres*, is a major fungal disease affecting barley, leading to significant yield losses globally. Improving the sustainability of barley production requires identifying genetic sources that confer effective resistance across genetic backgrounds and diverse environments. Considerable progress has been made through mapping studies conducted internationally, but many have largely focused on unadapted germplasm such as landraces or used foreign pathotypes. To support breeding outcomes in Australia, characterization of resistance alleles that are effective against local pathotypes is critical, along with investigating resistances that may already be present in breeding germplasm as this could reduce the time to deployment of resistant cultivars. In this study, we applied the local genomic estimated breeding value approach to perform haplotype mapping using a multi-reference parent nested association mapping (MR-NAM) population developed using resistant breeding lines as donor parents. The population was evaluated across three seasons in Queensland to investigate the genetic architecture of NFNB resistance and genotype-by-environment interactions. We identified 30 haploblocks, with seven representing novel genomic regions not previously associated with NFNB resistance. Three haploblocks on chromosomes 2H, 6H, and 7H were consistently associated with resistance across all environments, suggesting that they likely harbor stable resistance loci. Haplotype stacking analysis demonstrated that combining multiple resistance haplotypes significantly reduced disease severity, with lines carrying 6–10 resistance haplotypes showing progressive improvements in NFNB resistance. The distribution and effectiveness of these key haplotypes across environments, and in diverse genetic backgrounds adapted to Australia (Commander, Compass and La Trobe), highlight the potential to harness them in ongoing pre-breeding and breeding programs.

**Supplementary Information:**

The online version contains supplementary material available at https://doi.org/10.1007/s00122-026-05328-0.

## Introduction

The past few decades, innovation in agricultural practices and plant breeding has significantly enhanced crop productivity. However, projections indicate that the present rate of genetic improvement in cereals may not be sufficient to satisfy the dietary requirements of an expanding global population by 2050 (Tilman et al. 2011). Small grain cereals, including barley (*Hordeum vulgare* L.), rice (*Oryza sativa* L.), rye (*Secale cereale* L.), and wheat (*Triticum aestivum* L.), provide approximately 50% of the global food supply (Chen et al. 2026; Verma et al. 2022). Barley serves primarily as animal feed and is a crucial ingredient for beer production globally, ranking as the fourth most significant cereal crop globally (Verma et al. 2022).

Net blotch, caused by *Pyrenophora teres*, is one of the most economically damaging diseases affecting barley worldwide. Net blotch manifests in two distinct forms: the net form (NFNB), attributable to *P. teres f. teres* (*Ptt*), and the spot form (SFNB), caused by *P. teres* f*. macu.lata* (*Ptm*) (Smedegård-Petersen 1971). In susceptible cultivars, both forms can cause substantial yield losses of 10–40% (Tomić et al. 2024), with losses approaching 100% under severe conditions. Both forms occur in most major barley production regions globally, with their prevalence fluctuating annually. In Australia, net blotch is widespread and favored by cool, moist conditions, with the pathogen surviving on infected residues, seed and undergoing repeated infection cycles during the growing season (Martin et al. 2020; Richards et al. 2017). Barley is most vulnerable from tillering to grain filling, particularly under early infection. NFNB produces elongated, net-like lesions, whereas SFNB causes discrete dark spots with chlorotic halos, reflecting differences in pathogen–host interactions (Clare 2022). Resistance to NFNB and SFNB is controlled by multiple QTL across the barley genome, including major loci (*Rpt* for NFNB and *Spt* for SFNB) on chromosomes 1H–7H. For example, *Rpt1* (1H), *Rpt2* (2H), and *Rpt5* (6H) confer NFNB resistance, while *Spt1/Spt2* loci on 5H are associated with SFNB resistance (Clare 2022; Richards et al. 2017). In Australia, NFNB screening is commonly conducted using locally relevant isolates to ensure disease evaluations reflect prevailing pathogen populations. The *P. teres f. teres* isolate NB85 has been widely used in the previous studies and is representative of Australian pathogen diversity, making it suitable for assessing resistance under locally relevant conditions (Martin et al. 2020; Richards et al. 2017).

Genome-wide association studies (GWAS) and biparental mapping are widely used approaches for identifying trait-associated loci in crops. Biparental populations provide high power to detect loci segregating between two parents but are limited by low allelic diversity and recombination events. In contrast, GWAS utilizing diverse germplasm panels capture multiple alleles per locus and benefit from higher historical recombination, resulting in improved mapping resolution (Rafalski 2010). However, GWAS can be affected by population structure and uneven allele frequencies, which may reduce statistical power or lead to false-positive associations. Recent advances in barley genomics, including high-density SNP arrays (e.g., 50 K platforms), whole-genome sequencing, and pangenome resources, have further enhanced the ability to detect trait-associated loci with greater precision (Mascher et al. 2017; Mayer et al. 2012). These developments provide improved genome coverage and resolution compared to earlier genotyping platforms such as the 9K iSelect array.

Nested association mapping (NAM) populations combine the advantages of both biparental and association mapping approaches (McMullen et al. 2009; Nice et al. 2016). NAM populations comprise multiple alleles, with mating designs that enrich rare alleles, enabling QTL mapping for beneficial agronomic traits from diverse sources (Nice et al. 2017). Recent studies using GWAS and NAM approaches have identified novel alleles at both known and unknown loci affecting rust resistance, flowering time, plant development, stress response, and NFNB resistance (Martin et al. 2020; Maurer et al. 2015; Roy et al. 2025; Saade et al. 2016; Vatter et al. 2017).

Previous QTL mapping and GWAS studies have primarily identified single-marker associations using low to moderate density genotyping platforms. While useful for broad genomic region discovery, this method ignores the association between nearby alleles via linkage disequilibrium (LD) and hence has disadvantages when utilized in marker-assisted selection strategies (Meuwissen et al. 2001; Voss-Fels et al. 2019). An alternative haplotype-based mapping approach counteracts these limitations, where markers are grouped together based on LD and their cumulative effect calculated, and thus representative of the future chromosomal block inheritance in breeding. The use of the local genomic estimated breeding values (local GEBVs) serves as an example of this haplotype-based approach (Voss-Fels et al. 2019). Although substantial progress has been made in understanding the genetic basis of NFNB resistance in barley, most studies have used non-Australian isolates and germplasm. In Australia, only a limited number of studies have identified NFNB resistance QTLs on chromosomes 1H, 2H, 3H, 4H, 6H, and 7H using local pathotypes and germplasm (Lehmensiek et al. 2007; Martin et al. 2020). However, these studies were based on specific populations and environments and did not apply haplotype-based analysis or study multi-reference NAM populations. For effective resistance breeding, identifying haplotypes that confer stable resistance across environments is a high priority. The objectives of this study were to (1) explore genotype-by-environment (G × E) interactions for NFNB in Australian barley germplasm evaluated in a multi-environment trial, (2) identify resistance-associated haploblocks using the local GEBV approach, and (3) evaluate stacking of resistance haplotypes for enhanced NFNB resistance. It is anticipated these findings will inform breeding strategies for developing barley cultivars with stable NFNB resistance.

## Material and methods

### Plant material

For the multi-reference nested association mapping (MR-NAM) population, three Australian spring barley cultivars were selected as reference parents based on their widespread cultivation and industry preference. Commander, Compass, and La Trobe are cultivated extensively in the eastern, southern, and western production regions of Australia, respectively (Clare 2022; Martin et al. 2020; Richards et al. 2017). Compass and Commander are industry-preferred malting cultivars, while La Trobe, classified as a food-grade cultivar, is frequently exported for international malting markets. A mixture of other cultivars and elite breeding lines from the Northern Region Barley Breeding Program (NRBBP) were used as founder parents. The founder parents were selected for a range of biotic and abiotic traits, and the reference parents were selected based on industry and breeding program preference. Pure seed for all parent lines and NAM progeny was provided by the NRBBP.

### Population development

Using an incomplete crossing scheme, the 40 founder lines (C07.276 > DH049, NRB090257, NRB090885, NRB091087, NRB11116, NRB11713, NRB11755, NRB120142, NRB120554, NRB120567, NRB120742, NRB120834-4, NRB120850, NRB120862, NRB120883, NRB120970, NRB121137, NRB130200, NRB130293, NRB130715, NRB130777, NRB130788, NRB130790, NRB130851, NRB130855, NRB130856, NRB130868, NRB130958, NRB130978, NRB130990, NRB130999, NRB131116, NRB131137, NRB131153, NRB131183, NRB131201, NRB131322, NRB131326, Oxford, Westminster) were crossed to each of the three reference varieties (Compass, Commander, and La Trobe), producing 32 F_1_ crosses in total (Supplementary Fig. S1). F_1_ plants were advanced under speed breeding conditions at The University of Queensland (UQ) to rapidly generate large segregating F_2_ populations (Watson et al. 2018). F_2_ populations were sown as spaced plants in the field at either Wellcamp, or Warwick in QLD during the 2014 season and selected for agronomic traits (plant height and maturity) matching the respective reference parent. From each cross, a single spike containing 50–60 individual F_2_ plants was manually harvested. A single seed from each spike was subsequently grown under speed breeding conditions to generate F_2_:F_3_ lines. Single seed descent was continued to produce F_4_ plants. Selfed seed from each F_4_ plant was bulked to generate the F_4_:F_5_ lines comprising the final population (Supplementary Fig. S2).

### Screening for net form net blotch resistance

The MR-NAM population, comprising 2559 genotypes, was evaluated for NFNB resistance under field conditions at the Department of Primary Industries, Hermitage Research Facility, Warwick, QLD, Australia, during the 2016, 2017, and 2018 growing seasons. Approximately 15 seeds of each genotype were sown as a hill plot, spaced 50 cm from neighbors along the row, 76 cm and 21 cm across the row. Dash is a very susceptible barley cultivar, which was planted 2 weeks before the experimental hill plots in the rows between the MR-NAM materials to spread the disease evenly throughout the NFNB nursery to facilitate localized dispersal of conidia. The population was assessed using a resolvable incomplete block design with two replicates across all years.

A single *Pyrenophora teres f. teres* isolate (NB85) was used for all evaluations which enabled a focused evaluation of G × E effects across the seasons. The isolate was cultured on modified Czapek’s medium containing 0.5 g/L KH₂PO₄, 0.5 g/L MgSO₄, 0.5 g/L KCl, 1.2 g/L urea, 20 g/L lactose, and 20 g/L agar. Single-spore cultures were incubated under near-ultraviolet light with a 12-h photoperiod at 18–20 °C. Conidia were harvested by adding distilled water to culture plates and gently scraping the surface with a spatula. The suspension was filtered through two layers of cheesecloth to remove mycelial fragments and adjusted to 5000 conidia/mL. The harvested conidia (1 *µ*ml suspension) count was confirmed using a hemocytometer. Inoculation for phenotyping experiments followed a two-stage process. In the first stage, a 500 m^2^ field of a susceptible cultivar (Dash) was sown as a pre-season disease increase block. Spreader blocks were mist irrigated before inoculation, and approximately 12 patches (3 m^2^ each) were sprayed at sunset, covered with a tarpaulin overnight, and irrigated the following morning to promote disease development. Heavily infected plants developed after 2 months. In the second stage, infected plants were cut and transferred to the disease nursery, where infected straw was spread over spreader rows of susceptible plots. Sprinkler irrigation was applied regularly to promote disease development. Disease assessment was conducted approximately 6 weeks after the initial inoculation. Disease severity was assessed using a 1–9 scale, 1–3 (resistant), 4–6 (moderately susceptible), and 7–9 (susceptible) (Meldrum et al. 2004). Assessments were conducted when sufficient disease differentiation was observed. At least two assessments of disease response data were collected in each year and to compare the susceptibility levels of genotypes. The disease assessment days varied due to unfavorable weather conditions and insufficient disease response.

### DNA extraction and genotyping

Genomic DNA was extracted from bulked young leaf tissue using the CTAB protocol recommended by Diversity Arrays Technology (DArT™) (http://www.diversityarrays.com). DNA samples from the MR-NAM population were genotyped using the “Barley GBS 1.0” platform developed by DArT Pty Ltd, Canberra, Australia, as described by Li et al. (2015) to generate marker data from DArTseq™ single-nucleotide polymorphisms (SNPs).

### Multi-environment trial analysis

Data analysis was conducted using R (version 4.2.2, R Core Team 2022). Multi-environment trial analysis was performed to investigate genotype-by-environment (G × E) interactions using the “ASReml-R” package (Butler et al. 2009). The following mixed model was used:

$$\begin{aligned} {\boldsymbol{y}} = & {\mathbf{X}}{\boldsymbol{\tau}} + {\mathbf{Z}}{\boldsymbol{u}} + {\boldsymbol{e}} \\ = & {\mathbf{X}}{\boldsymbol{\tau}} + {\mathbf{Z}}_{{\mathbf{0}}} {\boldsymbol{\mu}}_{{\mathbf{0}}} + {\mathbf{Z}}_{{\boldsymbol{g}}} {\boldsymbol{\mu}}_{{\boldsymbol{g}}} + {\boldsymbol{e}} \\ \end{aligned}$$

where **X** and **Z** are the design matrices corresponding to the fixed effects (***τ***) and random effects (${\boldsymbol{u}}$).

To determine the optimal model for the G × E interactions, diagonal and correlation models with homogeneous and heterogeneous variance assumptions were fitted. A factor analytic (FA) variance structure was selected for the G × E interactions when genetic variances and covariances were heterogeneous among environments (Smith et al. 2001). Model selection was based on REML log-likelihood, AIC, and total genetic variance. The genotypic effect and variance were determined using the following equation:

$$\mu_{{\boldsymbol{g}}} = ({\boldsymbol{\lambda}}_{{\mathbf{1}}} \times {\mathbf{\ell }}_{{\boldsymbol{m}}} ){\boldsymbol{\mathpzc{f}}}_{{\mathbf{1}}} + \ldots + ({\boldsymbol{\lambda}}_{{\mathbf{4}}} \times {\mathbf{\ell }}{\boldsymbol{m}}){\boldsymbol{\mathpzc{f}}}_{{\mathbf{4}}} + {\boldsymbol{\delta}}$$

where δ is the vector of residuals, ${\mathbf{\ell }}_{{\boldsymbol{m}}}$ is the genetic variance matrix, and the coefficient λ is the known trial site loadings (Smith et al. 2001).

### Marker data curation and population structure analysis

Genome-wide DArTseq SNP markers were obtained for the MR-NAM population. SNP positions were based on the barley reference genome Morex v1.0 assembly. Of 11,122 polymorphic DArT markers identified, 9265 SNPs were mapped to the seven chromosomes, with the lowest marker coverage on chromosome 4H and the highest on 2H (Supplementary Fig. S3). SNP alleles were coded as 0, 1, and 2, corresponding to homozygous reference (AA), heterozygous (AT), and homozygous alternate (TT) genotypes, respectively. All SNP markers were filtered to eliminate potential errors in allele calls of the SNP markers with > 10% missing data and < 1% minor allele frequency, and individuals with excessive missing data (> 20%) were removed, yielding 7014 high-quality markers.

Population structure was analyzed using the R package “Selection Tools”. Genetic distance among all genotypes was calculated using 7014 SNPs via pairwise Roger’s distances. Principal coordinate analysis-based k-means clustering was applied, initially testing *k* = 15 subgroups. The optimal cluster number was determined using the R package “NbClust version 3,” which evaluates multiple clustering criteria. Cluster number was selected based on the highest number of indices, considering the first three principal components.

### Haplotype mapping and stacking analyses

Resistance-associated haploblocks were identified using the local GEBV approach (Shaffer et al. 2026; Voss-Fels et al. 2019). SNP effects were estimated simultaneously using a ridge regression best linear unbiased prediction (RRBLUP) model (Meuwissen et al. 2001). Linkage disequilibrium (LD) blocks were created using the R package “Selection Tools”. Using a marker tolerance of 3 and an LD threshold of *r*^2^ ≥ 0.4, a total of 2,964 LD blocks were identified. Marker tolerance represents the number of SNPs that can be incorporated despite being below the LD threshold. Haplotypes were defined as SNP combinations within each LD block. Overall haploblock effects were calculated by summing estimated allelic effects of markers within each block using the local GEBV method. The variance of effects for each haploblock was estimated, and a scaled min–max variance (sc_var) was used to rank haploblocks. The top 1% of haploblocks were selected as high-variance regions to define block significance. A well-maintained open-source R package is now available for performing local GEBV analyses called "HapSelect" (Shaffer et al. 2026). The physical positions of top haploblocks were subsequently converted from the Morex v1 to the Morex v3 barley reference genome to facilitate comparison with previously reported resistance loci. Colocation analysis was performed by comparing the physical positions of significant haploblocks with published NFNB resistance QTL and genes, with the co-located regions summarized in Table 2.

Haplotype stacking analysis was conducted to explore additive effects of multiple haplotypes. This involved determining the number of beneficial haplotypes per genotype for high-variance blocks and investigating their cumulative effect on disease resistance. For this analysis, haploblocks with a scaled variance > 0.2 were selected. Haplotypes with positive effects (increased susceptibility) were excluded. Genotypes carrying only negative effect (resistance-associated) haplotypes within high-variance blocks were identified, and the cumulative effect of stacking multiple resistance haplotypes on disease severity was assessed.

## Results

### Net form net blotch disease severity across environments

**Fig. 1:**
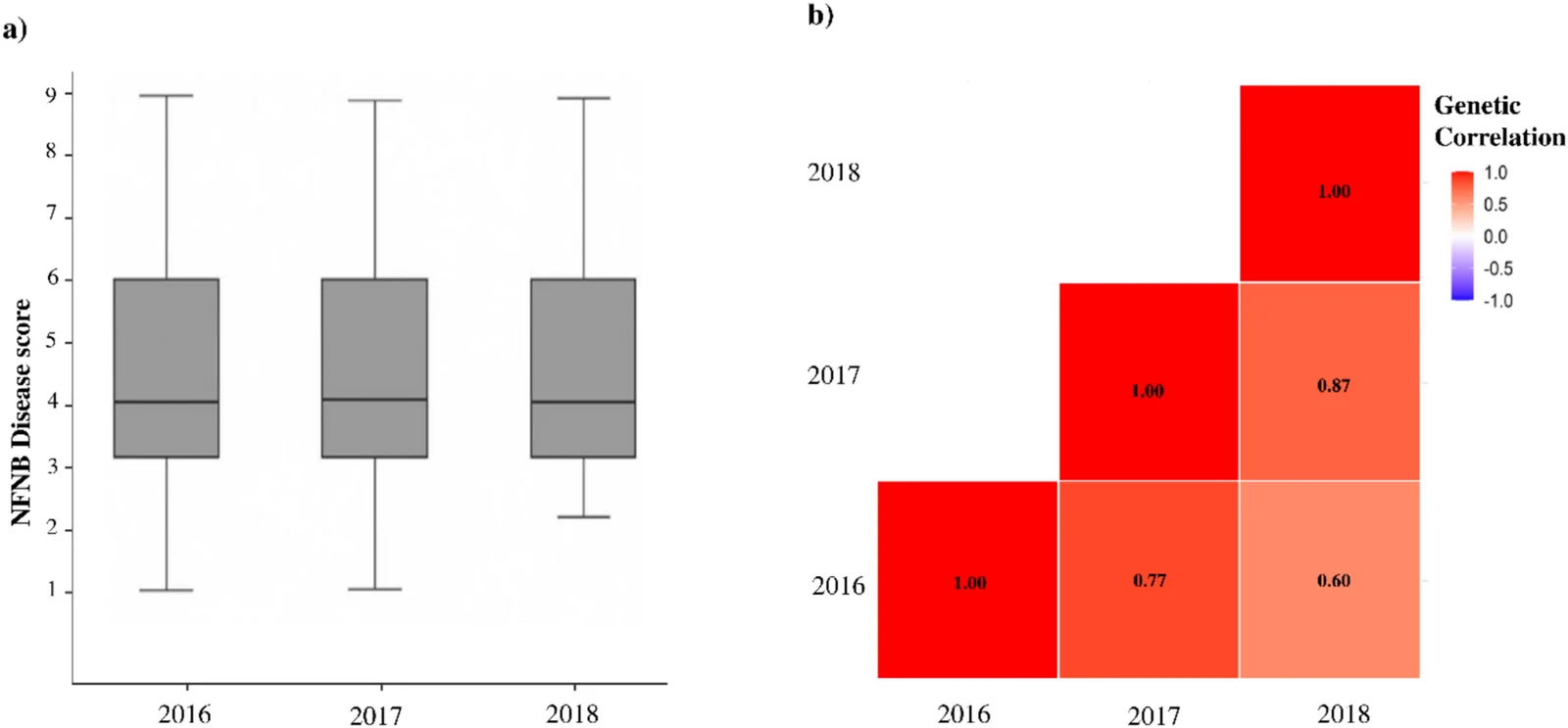
Variation in NFNB disease severity and genetic correlations in the MR-NAM population. **a** Box plot showing distribution of raw disease severity scores on a scale from 1 to 9 across 3 years. **b** Genetic correlations between environments across all 3 years using the factor analytic 1 (FA1) model

Disease severity exhibited a wide range of phenotypic variation over the 3-year evaluation period. Disease severity scores ranged from 1 to 9 in both 2016 and 2017, and from 2 to 9 in 2018 (Fig. 1a; Supplementary Table S1). The distribution of phenotypes remained consistent across the years, indicating reliable disease pressure and screening conditions. Genetic correlations between environments confirmed the presence of G × E interactions (Fig. 1b). The highest correlation (0.87) was observed between 2017 and 2018, while 2016 showed moderate correlations with both 2017 (0.77) and 2018 (0.60), confirming significant G × E interactions within the dataset.

Model fitting revealed substantial crossover and scale-type G × E interactions in genotype rankings across environments (Supplementary Fig. S4). Progressive application of increasingly complex variance structures, from diagonal to factor analytic 1 (FA1), showed that FA1 provided the best fit based on AIC, BIC, and log-likelihood values, accounting for 82.68% total genetic variance (Table 1).

**Table 1:** Model comparison for variance structures fitted in the multi-environment trial analysis using Akaike Information Criteria (AIC), Bayesian Information Criterion (BIC), and Log-likelihood (LogLik) statistics

Model	AIC		BIC		LogLik		%Variance accounted	
diag		11,403		11,525		− 5683.588		NA
corv		10,346		10,427		− 5160.813		NA
corh		10,042		10,137		− 5006.951		NA
FA1		9,965		10,121		− 4959.221		82.68

### Population structure of the MR-NAM population

**Fig. 2:**
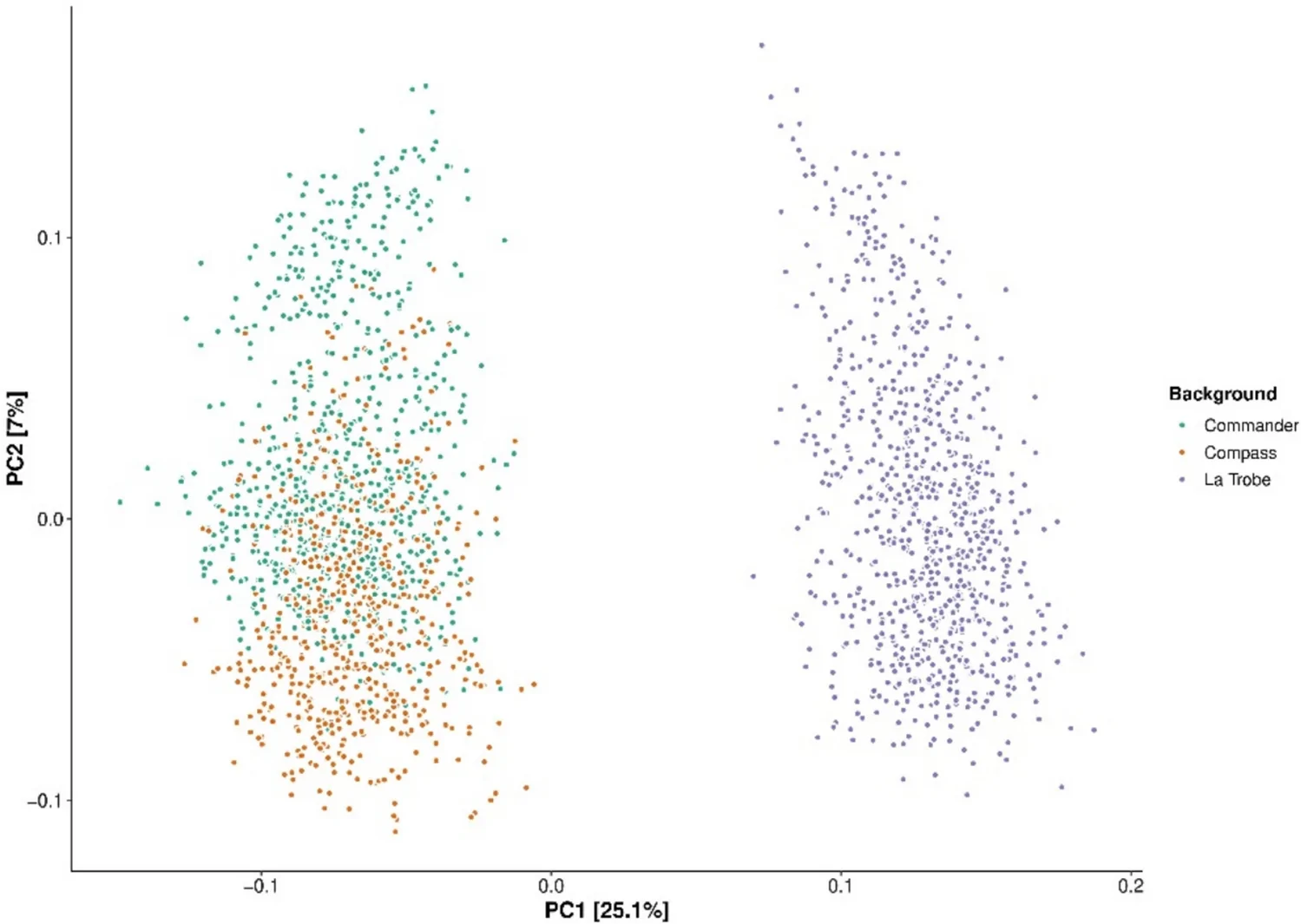
Principal component analysis (PCA) of the MR-NAM population structure. The analysis was based on pairwise Rogers’ distances calculated from 7014 high-quality polymorphic SNP markers across 2559 MR-NAM lines. PC1 explained 25.1% of the variance and PC2 explained 7%

Principal component analysis (PCA) of the MR-NAM population revealed two main clusters. The first principal component (PC1) explained 25.1% of the total variation, while the second principal component (PC2) accounted for 7% (Fig. 2). Cluster 1 consisted of 870 lines derived from the La Trobe parent, whereas Cluster 2 comprised 837 lines from Commander and 738 lines from Compass. La Trobe formed a distinct cluster, while Commander and Compass clustered together, reflecting their close genetic relationship.

### Local GEBV analysis identifies 30 haploblocks associated with NFNB severity

**Fig. 3:**
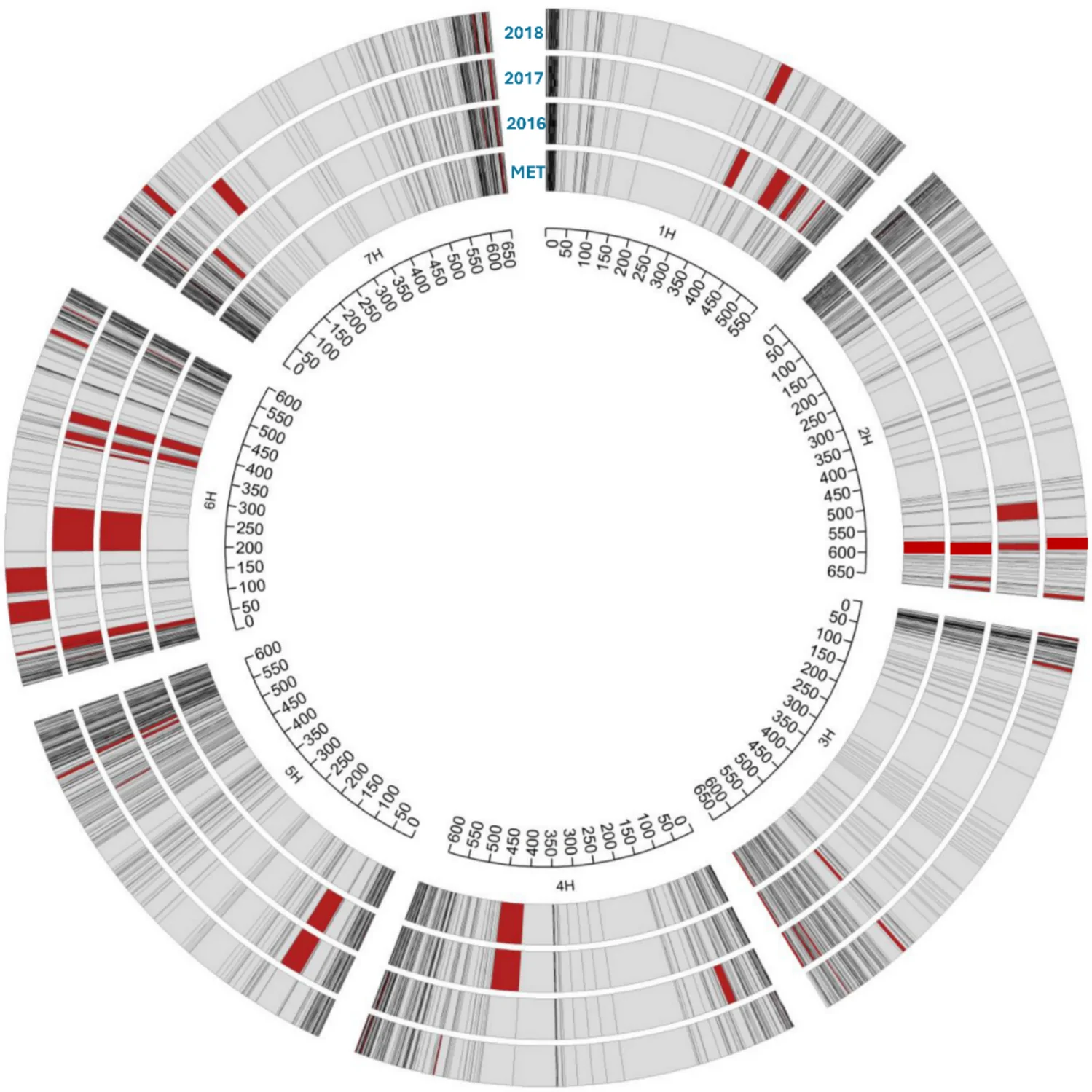
Circular plot showing scaled haploblock variance for 2016, 2017, 2018, and MET. Red color represents top 1% high-variance haploblocks and their position according to Morex v3 (Mbp)

A total of 30 high-variance haploblocks associated with NFNB severity were identified across the three individual years (2016, 2017, and 2018) and MET analysis (Fig. 3; Table 2; Supplementary Table S2). Seventeen haploblocks were unique to a single analysis, while thirteen appeared in multiple analyses. Three haploblocks on chromosomes 2H (578.3–582.4 Mbp), 6H (43.1–46.9 Mbp), and 7H (617.2–619.8 Mbp) exhibited high variance across all four analyses, indicating consistent and stable effects. Additionally, two haploblocks on chromosomes 3H and 5H showed high variance in 2016, 2017, and MET analyses. These findings highlight key genomic regions influencing NFNB severity and provide valuable targets for breeding programs.

**Table 2:** Summary of high-variance haploblocks for NFNB disease response and their colocation with known QTLs or genes

Block	Chr^a^	Start position(bp)^b^	End position(bp)^b^	Block size (bp)	Variance				*NFNB* gene(s)/QTL within haploblock	References
					2016	2017	2018	MET		
b000125	1H	328,645,535	343,982,957	15,337,422	0.286	NA	0.273	NA	*None*	
b000139	1H	394,002,083	417,000,622	22,998,539	0.727	NA	NA	NA	*QRptts-6H-175.80 in close proximity, SCRI_RS_138118*	Adhikari et al. (2020), Richards et al. (2017)
b000257	1H	506,489,231	506,489,299	68	NA	NA	0.196	NA	*QRptts-1H-92–93, QPt.ta-1H-1: 11_11189*	Afanasenko et al. (2015)
b000591	2H	455,668,994	478,374,210	22,705,216	NA	0.226	NA	NA	*None*	
b000606	2H	511,268,588	519,755,100	8,486,512	NA	0.265	NA	NA	*Bmag0114–p11m54T105*	Cakir et al. (2011)
b000637	2H	578,320,284	582,432,898	4,112,614	1	0.135	0.353	0.049	*Bmag0114*	Cakir et al. (2011)
b000740	2H	618,189,310	620,042,463	1,853,153	0.652	NA	0.636	NA	*JHI-Hv50k-2016–104508, JHI-Hv50k-2016–104859*	Rozanova et al. (2019)
b000771	2H	611,656,397	629,446,334	17,789,937	NA	0.204	0.270	NA	*JHI-Hv50k-2016–74407 in close proximity*	Rozanova et al. (2019)
b000866	2H	653,957,222	653,957,285	63	0.291	NA	NA	NA	*QRptts-2H-92.8–93.93, SCRI_RS_10670, SCRI_RS_128449, SCRI_RS_138463*	Adhikari et al. (2020)
b001041	3H	461,735,346	465,609,776	3,874,430	NA	NA	0.207	NA	*JHI-Hv50k-2016–16473*	Novakazi et al. (2019)
b001152	3H	559,417,074	562,155,599	2,738,525	NA	0.267	NA	NA	*HVM60*	Cakir et al. (2011)
b001133	3H	549,619,437	551,310,875	1,691,438	NA	NA	NA	0.104	*QTL_um-3H*	König et al. (2013)
b001173	3H	576,221,817	581,285,446	5,063,629	0.357	0.381	NA	0.219	*HVM60*	Cakir et al. (2011)
b001174	3H	581,635,584	585,721,403	4,085,819	NA	0.282	NA	NA	*QRptts-3H-64.60*	Adhikari et al. (2020)
b001357	4H	395,242,996	438,760,592	43,517,596	0.930	NA	NA	0.240	*QNFNBAPR.Al/S-4Ha, ABG3–ABG484*	Lehmensiek et al. (2007), Steffenson et al. (1996)
b001515	4H	596,215,352	597,601,046	1,385,694	NA	NA	0.224	NA	*QRptta-4H-81.57*	Amezrou et al. (2018)
b001568	5H	4,289,784	4,851,348	561,564	0.365	NA	NA	NA	*QRptm-5H-12–21*	Alhashel et al. (2023)
b001639	5H	63,221,468	91,590,062	28,368,594	0.446	0.262	NA	0.125	*Qns-5H*	Daba et al. (2019)
b002246	6H	37,790,284	42,205,809	4,415,525	NA	NA	NA	0.332	*QRptts-6H*	Amezrou et al. (2018)
b002248	6H	43,161,457	46,965,809	3,804,352	0.326	1	0.755	1	*None*	
b002263	6H	188,400,682	249,934,963	61,534,281	NA	0.437	NA	0.121	*Qns-6H*	Daba et al. (2019)
b002271	6H	340,307,090	352,939,420	12,632,330	NA	NA	NA	0.125	*Rpt-M8*	Richards et al. (2017)
b002273	6H	350,329,718	350,329,650	69	NA	NA	NA	0.111	*None*	
b002277	6H	362,306,329	374,039,957	11,733,628	0.585	NA	NA	0.250	*None*	
b002290	6H	388,190,265	399,996,664	11,806,399	NA	0.372	NA	0.285	*Qnfnb-6H*	Daba et al. (2019)
b002379	6H	533,400,700	534,888,685	1,487,985	NA	NA	0.313	NA	*QRptts-6H*	Amezrou et al. (2018)
b002616	7H	39,596,969	41,947,974	2,351,005	NA	NA	0.202	NA	*QRptta-7H*	Amezrou et al. (2018)
b002718	7H	156,649,590	170,473,560	13,823,970	NA	0.257	NA	NA	*None*	
b002938	7H	616,862,969	617,248,002	385,033	NA	NA	0.216	NA	*QRpt-7H.3*	Clare (2022)
b002939	7H	617,248,030	619,852,516	2,604,486	0.358	0.733	1	0.250	*None*	

^a^Chromosome

^b^Physical position in base pairs (bp), where chromosomal position based on the physical map of the barley cultivar Morex V3 version (Mascher 2020)

Twenty-three haploblocks contained previously identified NFNB QTLs distributed across all seven chromosomes (Table 2). In contrast, seven haploblocks on chromosomes 1H, 2H, 6H, and 7H contained no previously reported *Rpt* genes or QTLs, suggesting that they harbor novel resistance loci (Table 2).

To assess haplotype effects within each block, scaled variance graphs were generated (Supplementary Fig. S5). Most analyses showed a balanced distribution of positive and negative effect haplotypes, except for the MET, which was slightly skewed toward negative effects. Haploblocks with the highest scaled variances were selected for subsequent analysis.

### Stacking of resistance haplotypes reduces disease severity

**Fig. 4:**
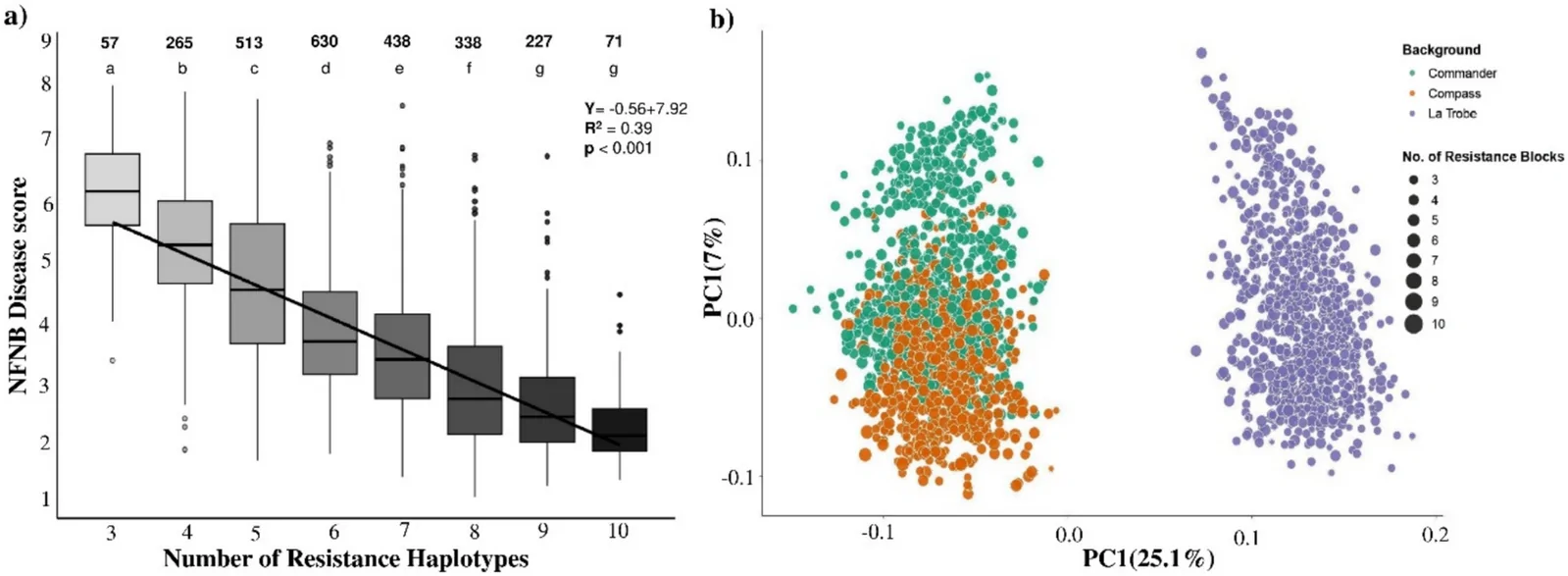
Haplotype stacking analysis of resistance haplotypes for NFNB. **a** Effect of resistance haplotype number on NFNB severity. Number on top of each bar represents the number of accessions carrying resistance haplotypes. **b** Distribution of genotypes in the MR-NAM population. Each point represents a genotype, colored according to its genetic background (Commander, Compass, or La Trobe). The size of each point corresponds to the number of resistance haplotype blocks carried by that genotype

The effect of carrying multiple resistance haplotypes on mean disease severity was evaluated. Figure 4a demonstrates a clear negative correlation between the number of resistance haplotypes and NFNB disease severity. Individuals with fewer resistance haplotypes (3–5) exhibit higher mean disease scores, indicating greater susceptibility, while those with more haplotypes (6–10) showed progressive reductions in disease severity, demonstrating the additive effect of haplotype stacking for conferring resistance. Greater variability in disease scores was observed among individuals with four or five resistance haplotypes compared to those with six or more, possibly due to genetic background effects or the limited number of individuals with higher haplotype counts. Individuals with high numbers of resistance haplotypes were distributed across the genetic diversity of the population and were present in all major genetic clusters (Fig. 4b). Individuals carrying multiple resistance haplotypes were distributed across diverse genetic clusters, indicating that NFNB resistance can be sourced from a range of backgrounds. This provides breeders with the flexibility to combine resistance haplotypes with other desirable traits, or resistance to additional diseases, by selecting donor lines from different populations.

**Fig. 5:**
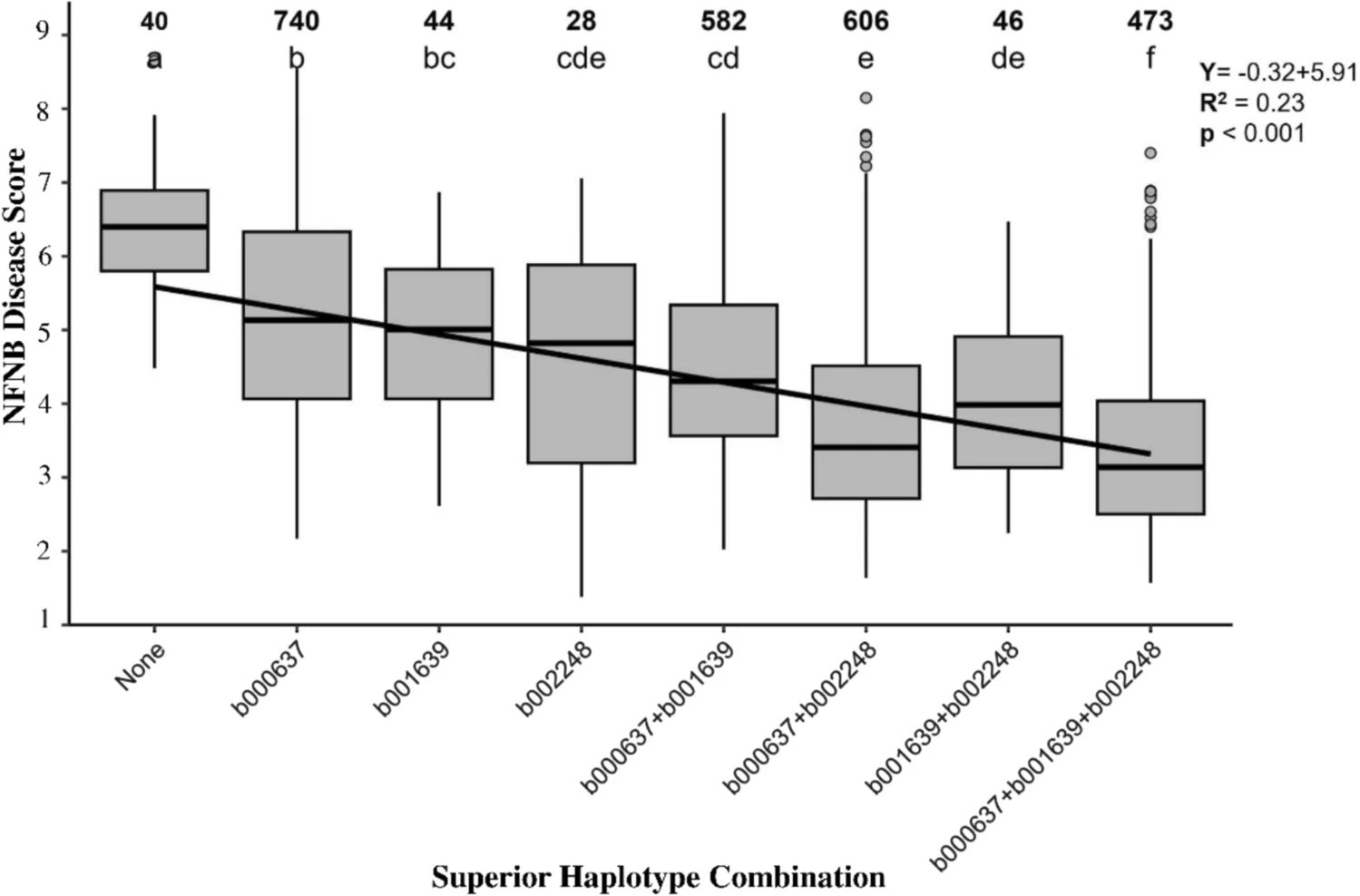
Boxplot illustrating the effect of superior haplotype combinations on NFNB disease severity. The y-axis represents phenotype values (disease score, where 9 is highly susceptible, and 1 is highly resistant), while the x-axis shows groups of NAM lines carrying none, single, or combination of multiple superior resistance haplotypes (2H:b000637, 5H:b001639, and 6H:b002248)

The distribution of disease severity across genotypes was considerably influenced by the presence and combination of superior resistance haplotypes. Genotypes lacking any superior haploblocks exhibited the highest severity. However, the presence of resistance haplotypes at key haploblocks such as 2H:b000637, 5H:b001639, or 6H:b002248 was associated with a reduction in disease severity (Fig. 5), where genotypes carrying all three superior haplotypes displayed the lowest disease response on average (Fig. 5). Notably, haploblocks 2H:b000637 and 6H:b002248 were identified in all environments and MET analysis; of which 6H:b002248 was deemed potentially novel (Table 2). This signifies an additive contribution of haplotype combinations and supports the approach of pyramiding resistance alleles to improve NFNB resistance levels.

## Discussion

### Phenotypic variation and environmental stability of NFNB resistance

NFNB is a significant foliar fungal disease that poses a serious threat to production across all major barley-growing regions globally. Although the disease can be managed through fungicide applications and appropriate agronomic practices, developing and deploying resistant cultivars remains the most economically viable and environmentally sustainable strategy. Therefore, identifying novel resistance genes is essential for breeding programs aimed at achieving durable NFNB resistance. Our phenotypic evaluation revealed substantial variation in NFNB response among barley genotypes, with resistance levels strongly influenced by environmental conditions and G × E interactions (Fig. 1). This variation is consistent with previous findings showing that elite barley breeding lines often carry partial resistance effective across years and locations, contributing to long-term disease control (Wang et al. 2015; Wonneberger et al. 2017a).

NFNB resistance expression was influenced by G × E interactions, emphasizing the importance of multi-environment testing for identifying stable resistance. While some resistance haplotypes were effective across all environments, others showed environment-specific effects. Certain haploblocks were detected only in specific years, consistent with previous reports of environment-dependent NFNB QTL. Wonneberger et al. (2017b) found that, aside from a consistent 5H locus, several QTL were significant in only one of the 3 years of field testing, likely due to environmental differences or pathogen variation. Similarly, Novakazi (2020) identified approximately 15 QTL across multiple locations, with some loci site specific or growth stage dependent. These studies demonstrate that breeding lines may appear susceptible or resistant depending on the test environment, complicating selection for broadly effective resistance.

Our results demonstrate that certain resistance-associated haploblocks were consistently identified across all 3 years, suggesting the presence of environmentally stable resistance loci. Conversely, haploblocks detected in single years likely represent environment-specific resistance mechanisms. The broad and repeatable phenotypic variation, with several lines consistently exhibiting resistance, suggests the presence of stable resistance loci within these genotypes. In contrast, monogenic resistances (such as certain *Rpt* genes) have often been overcome by new pathogen races or virulence alleles (Richards et al. 2017). The presence of genotypes with consistent, moderate resistance levels in the NAM germplasm is encouraging, as it indicates genetic factors that confer protection across environmental conditions.

### Haplotype mapping reveals novel resistance loci

Haplotype-based mapping proved powerful for identifying previously unknown NFNB resistance loci in Australian barley. By analyzing haploblocks (linkage disequilibrium-defined SNP clusters) and their variance in haplotype effects, we identified genomic regions associated with resistance (Fig. 3). This approach complements traditional GWAS, which often detects major-effect loci but can overlook polygenic or allele combination effects (Lorenz et al. 2010). Indeed, conventional GWAS of global barley panels have mainly rediscovered known NFNB resistance QTL corresponding to major genes (Daba et al. 2019). For instance, most marker–trait associations for NFNB in Ethiopian and ICARDA barley accessions overlapped with previously reported resistance loci, highlighting the challenge of discovering novel QTL through standard approaches (Daba et al. 2019).

Our focus on haploblocks with high variance allowed the detection of previously undetected resistance loci. These high-variance haplotypes likely capture beneficial allele combinations that individually have small effects but collectively confer significant NFNB resistance. Similar haplotype-based approaches in barley have revealed additional spot blotch resistance QTL on chromosomes 1H, 3H, and 6H that were missed by single-marker analysis (Roy et al. 2025). The effectiveness of haplotype mapping for QTL discovery is further supported by Vatter et al. (2017), who found six novel NFNB resistance QTL through nested association mapping of wild barley diversity. Our results support those findings, where several resistance-associated haploblocks identified here do not correspond to previously cataloged NFNB QTL in cultivated barley. This indicates that Australian barley breeding germplasm, particularly founders sourced from the NRBBP, harbor untapped resistance sources. Utilizing haplotype information (rather than individual SNPs) is key to identifying these novel loci, as it captures linkage relationships and rare alleles with collective efforts on resistance (Lorenz et al. 2010).

The superior haploblock combinations identified in this study provide further insight into the complex genetic architecture underlying NFNB resistance in barley. In particular, favorable alleles at 2H:b000637, 5H:b001639, and 6H:b002248 were associated with reduced disease severity. The colocalization of 2H:b000637 and 5H:b001639 with previously reported NFNB resistance regions supports the biological relevance of these haploblocks (Clare 2022; Richards et al. 2017). In contrast, 6H:b002248 did not clearly correspond to major previously reported loci and, therefore, may represent a potentially novel resistance-associated region in Australian barley germplasm. Gene annotation within this interval identified several defense-related candidate genes, including Protein AIG1, HSP70, ubiquitin carboxyl-terminal hydrolase, and multiple F-box proteins, which are known to play roles in plant immune responses and stress signaling pathways (Craig and Tyers 1999; Duplan and Rivas 2014; Wang et al. 2004). Although chromosome 6H is recognized as an important region for NFNB resistance, including the *Rpt5* region and other loci associated with the barley–*Pyrenophora teres* interaction (Clare 2022; Richards et al. 2017), the physical position of b002248 suggests that it may represent a distinct resistance locus. Therefore, this region represents a promising target for further fine-mapping and functional validation to confirm its role in NFNB resistance.

### Breeding applications: haplotype stacking and strategic deployment

Identifying multiple NFNB resistance haplotypes enables pyramiding approaches to develop barley cultivars with enhanced and durable resistance (Tong et al. 2024). Disease resistance often exhibits quantitative inheritance controlled by multiple small-effect genes distributed across the genome. Our haplotype analysis demonstrated that the combination of resistance haplotypes enhances resistance levels. A negative linear relationship between resistance haplotype number and disease severity indicated that lines carrying more resistance haplotypes displayed lower disease levels (Fig. 4). The previous studies have shown that resistance genes can interact additively or epistatically to enhance overall protection (Wiesner-Hanks and Nelson 2016). Haplotype-based mapping may more effectively capture these interactions, providing insights into optimal resistance gene combinations.

Both environmentally stable and environment-specific resistance types are important for breeding programs. Addressing G × E challenges requires identifying and combining QTL that contribute to resistance under different environmental conditions, thereby reducing G × E effects. Qian et al. (2017) highlighted that greater haplotype diversity can enhance trait stability across variable conditions, a principle equally applicable to disease resistance. Stable-effect loci are suitable for broad deployment, while environment-specific loci could be deployed strategically in regions with particular pathotypes and climatic conditions. Understanding G × E interactions in NFNB resistance is, therefore, essential for designing QTL combinations that integrate broad and targeted resistance, enhancing cultivar performance across diverse environments and pathogen populations.

The value of pyramiding is supported by Daba et al. (2019), who found that Ethiopian barley landraces carried resistance to both NFNB and leaf scald, representing natural gene stacks for multi-disease resistance. Practically, breeders should evaluate NFNB resistance under diverse conditions to identify haplotypes conferring consistent protection. Integrating multiple NFNB resistance sources into barley cultivars may provide a sustainable strategy for durable pathogen control. This study represents the first investigation of NFNB resistance using an MR-NAM population in barley, combining advanced mixed modeling of G × E interactions with haplotype-based mapping to comprehensively characterize genomic regions associated with both stable and environment-specific resistance. The haplotype mapping approach provides opportunities to enhance the barley resistance gene pool with loci that could strengthen genetic protection against NFNB beyond currently deployed resistance genes. Identifying and utilizing such resistances is crucial for developing sustainable, high-yielding barley cultivars for the future.

## Supplementary Information

Below is the link to the electronic supplementary material.

Supplementary file1 (PDF 496 kb)

Supplementary file2 (XLSX 333 kb)

Supplementary file3 (CSV 6 kb)

## Data Availability

The datasets generated during and/or analyzed during the current study are available from the corresponding author on reasonable request.
