# Supplementary material for: Haplotype-based nested association mapping for net form net blotch response in Australian barley
Source: Theor Appl Genet. 2026 Aug 18;139(9):233. doi: 10.1007/s00122-026-05328-0 (PMC13486095; doi:10.1007/s00122-026-05328-0)
Supplement: Supplementary file 1 [file 122_2026_5328_MOESM1_ESM.pdf]

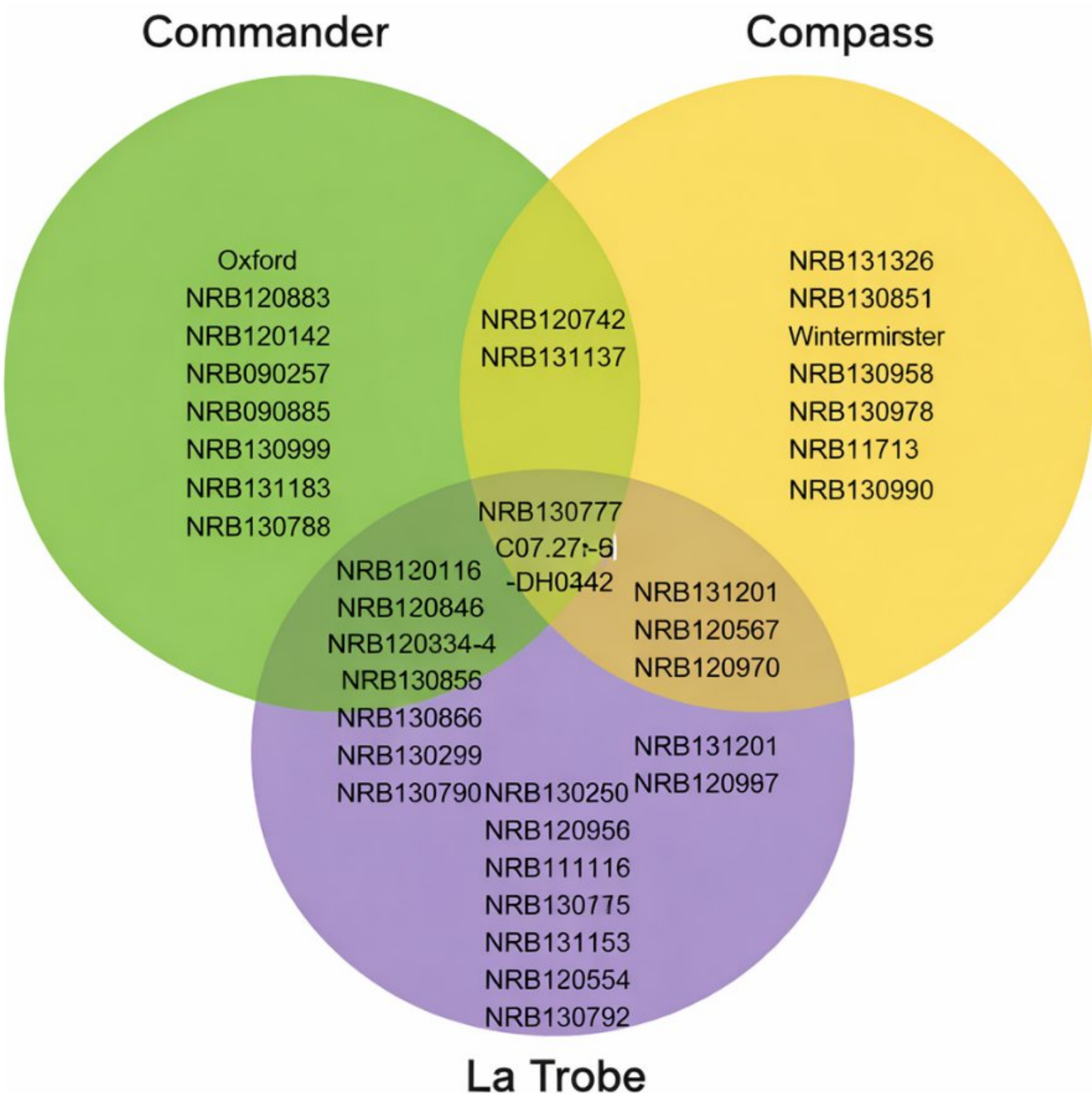

Supplementary Fig. S1: Venn diagram depicting the structure of the MR-NAM population, where each circle represents a reference variety. The donor parents crossed to the respective recurrent variety. The overlapping circle indicates that the founder has been crossed to more than one of the reference varieties. There are a total of 3 reference varieties and 40 donors.

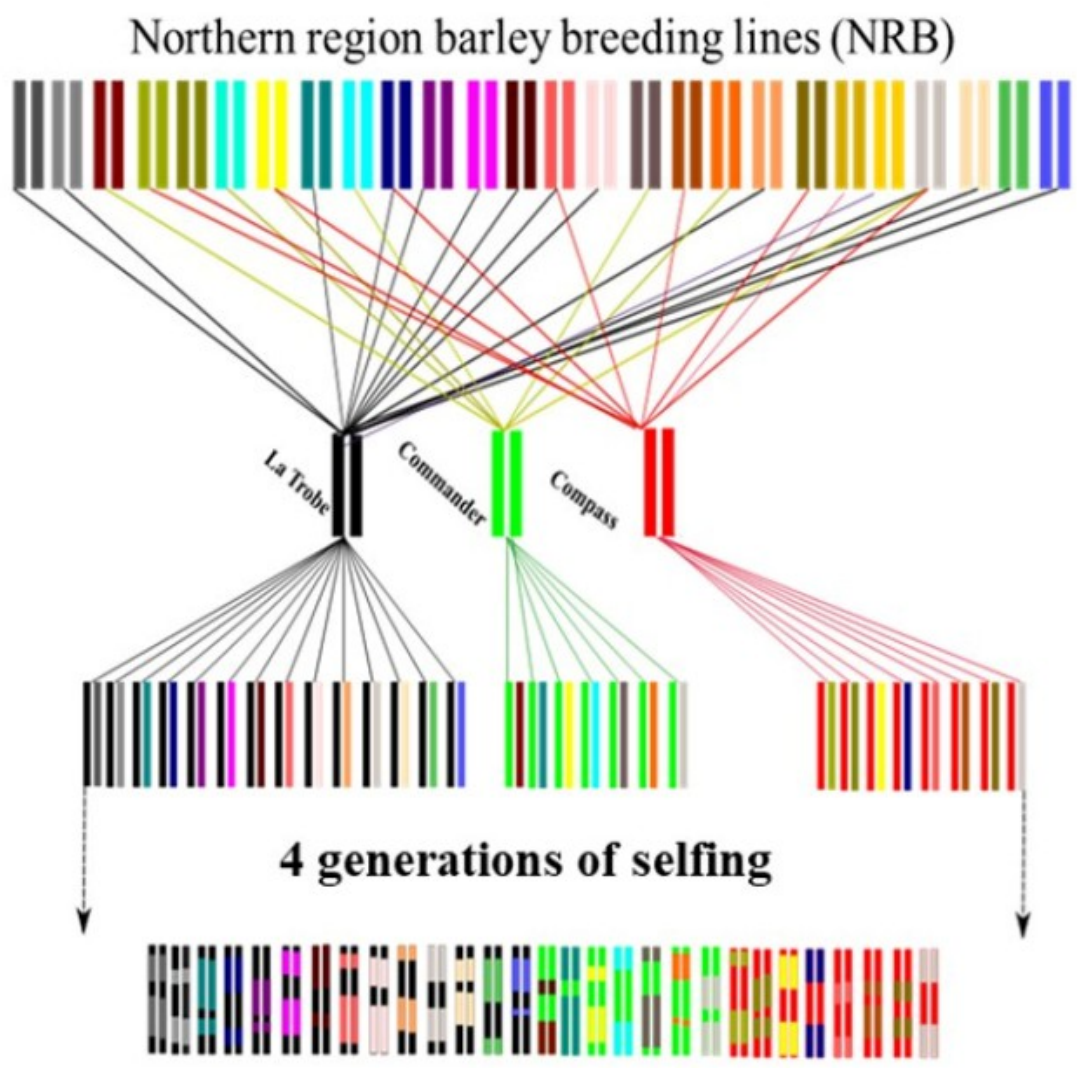

Supplementary Fig. S2: Schematic representation of the development of MR-NAM population.

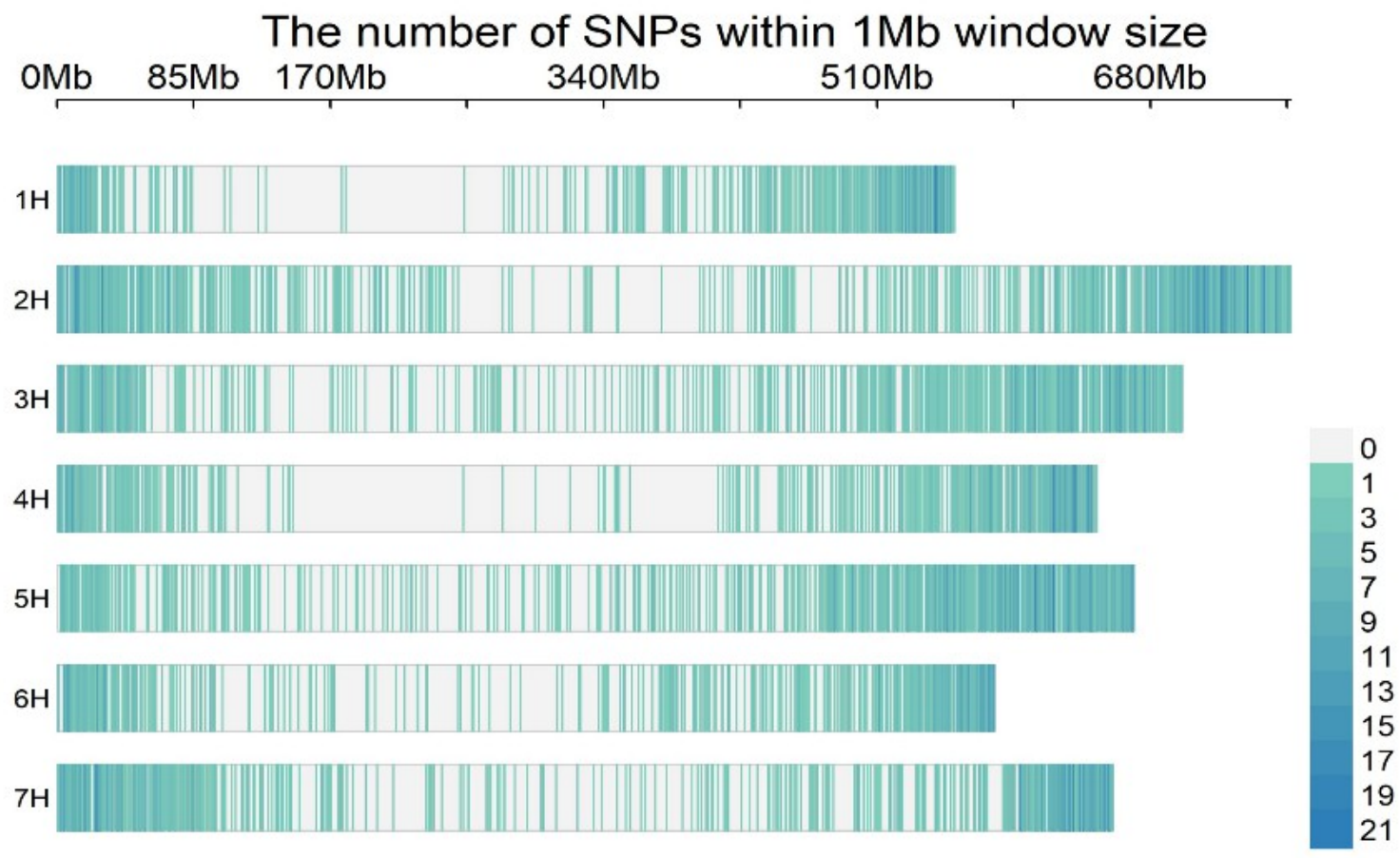

Supplementary Fig. S3: Marker density plot across the barley genome.

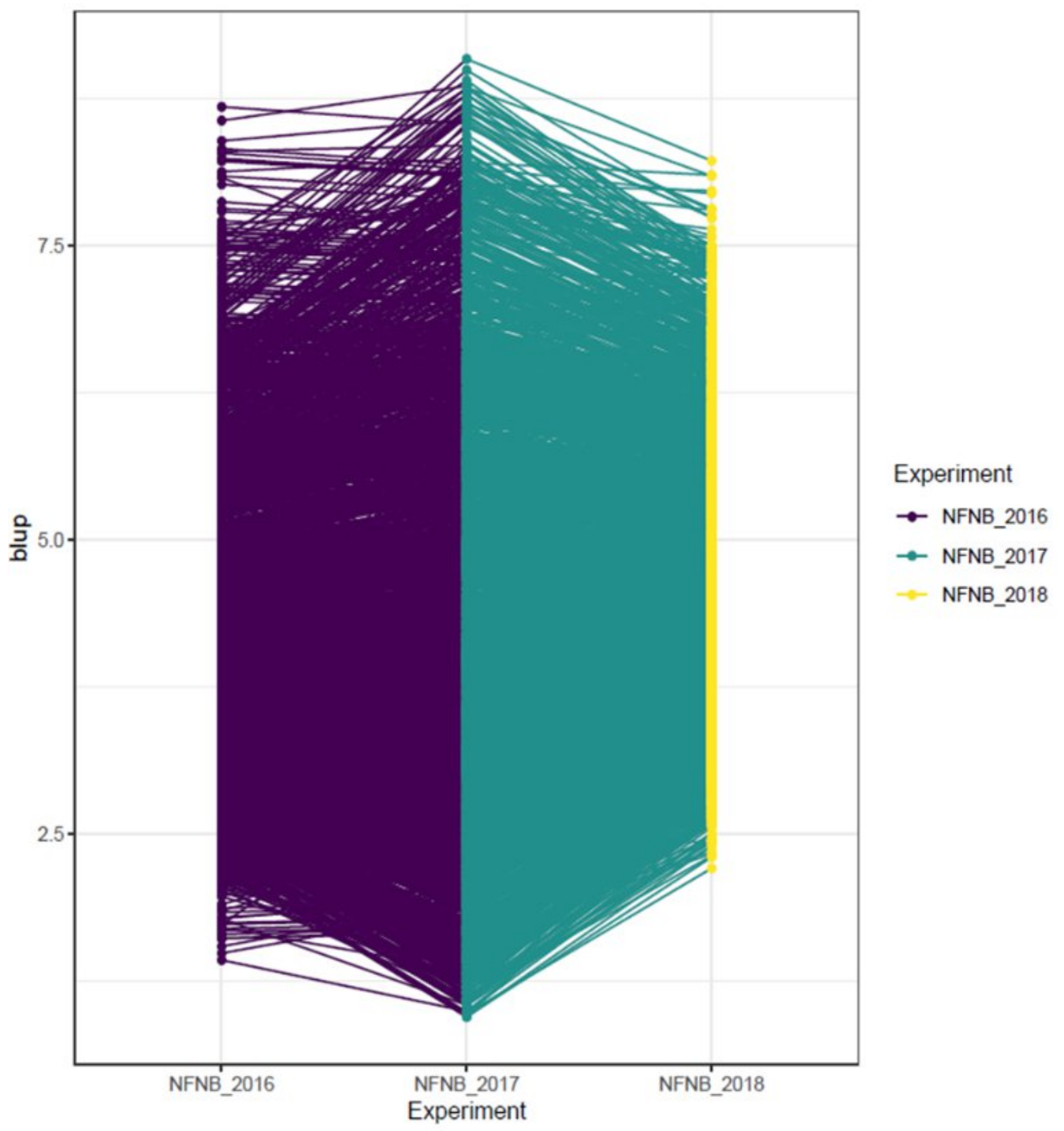

Supplementary Fig. S4: Crossover and G×E interactions in genotype rankings across environments.

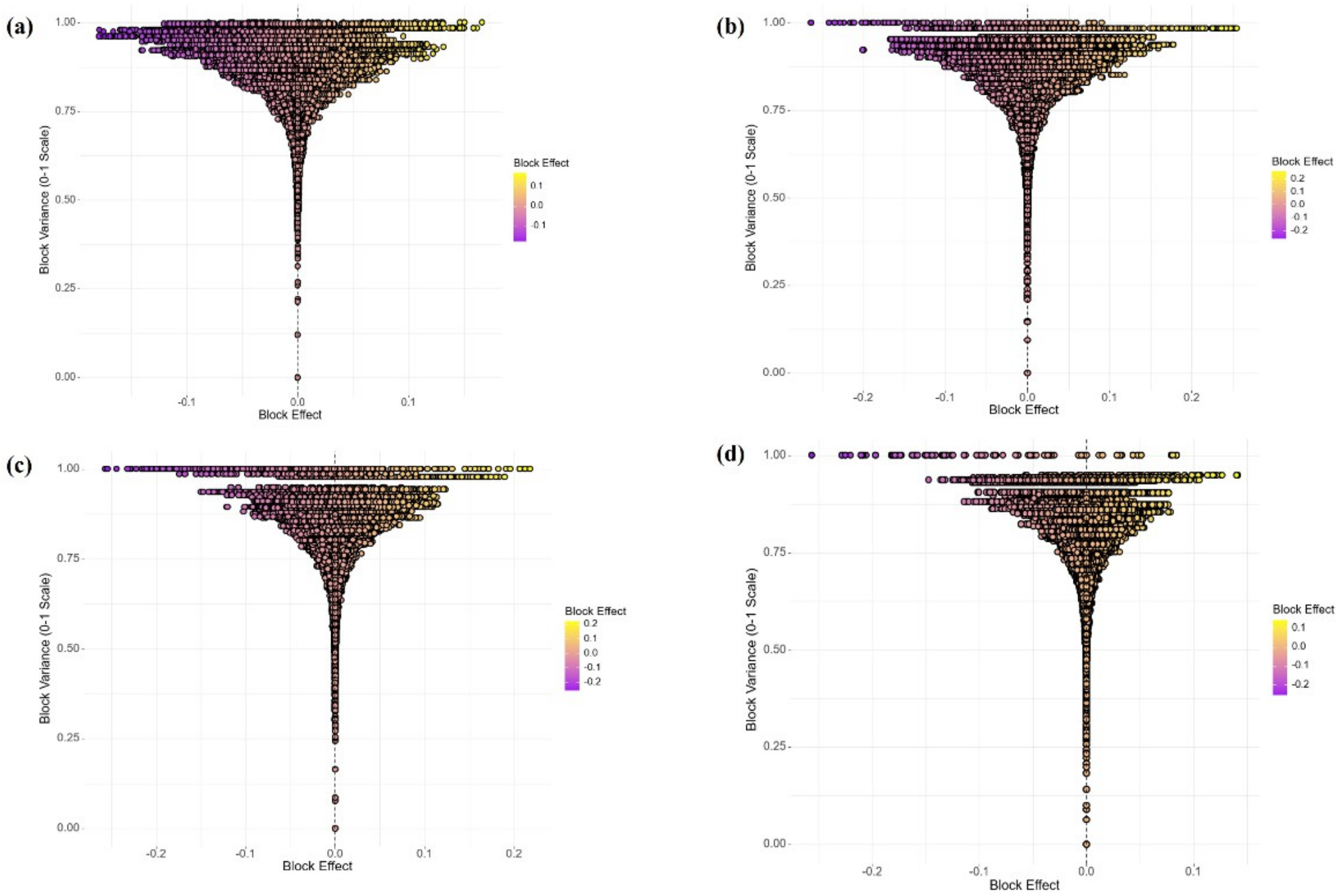

Supplementary Fig. S5: Association of scaled block variance with haplotype effects in (a) 2016, (b) 2017, (c) 2018, and (d) MET trials in the MR-NAM population.
